# Negative life events and depression among Chinese university freshmen: a longitudinal moderated mediation model

**DOI:** 10.3389/fpubh.2024.1480394

**Published:** 2024-11-13

**Authors:** Pei Jiang, Zhihui Zhang, Shuqin Li, Yuan Yan

**Affiliations:** ^1^School of Educational Science, Hunan Normal University, Changsha, Hunan, China; ^2^Department of Mental Health Education and Counseling Center, Hunan Institute of Engineering, Xiangtan, Hunan, China; ^3^Clinical Psychology Department, Alliant International University, Emeryville, CA, United States

**Keywords:** negative life events, depression, freshmen, perceived social support, self-esteem

## Abstract

**Objectives:**

Depression is a major mental health concern among university freshmen, with negative life events recognized as a risk factor. However, limited studies have explored the underlying mechanisms between them. This study aims to investigate the longitudinal relationship between negative life events and depression, and to examine both the potential mediating role of perceived social support and the moderating role of self-esteem in this process.

**Methods:**

A two-wave longitudinal design with a 6-month interval was employed, involving 785 Chinese university freshmen (55.92% were female). Self-reported measures of negative life events, perceived social support, self-esteem, and depression were collected using validated scales.

**Results:**

Results revealed that negative life events significantly predicted depression in freshmen after 6 months, with perceived social support partially mediating this relationship. In addition, self-esteem moderated the effect of negative life events on perceived social support. As self-esteem increased, there was a stronger mediating effect of perceived social support between negative life events and depression.

**Discussion:**

This study verified a moderated mediation process of how negative life events impact depression among freshmen. The findings provide valuable insights for developing effective interventions to reduce the depression risk of freshmen.

## 1 Introduction

Depression has a significant impact on individual physical and mental health, leading not only to a decline in quality of life but also to the burden of disability and disease (1, 2). In 2022, the National Depression Blue Book Survey in China shows that the prevalence of depression among adolescents has reached 15–20%. Among them, 50% of the patients with depression were students in school. Due to depression, 41% of the students have stopped going to school (3). With the increasing prevalence of depression in China in recent years, the National Health Commission of the People's Republic of China issued a plan for the prevention and treatment of depression in 2020, highlighting the urgent need for intervention among university students (4). Particularly during the transition period, university freshmen face numerous changes, such as building new social networks, adapting to academic pressures, and managing personal finances. These adjustments can be challenging as they move from a supportive and structured environment to an independent and self-directed one. Research has shown that the first year of college carries a high risk of developing depression-related symptoms (5). Moreover, the overall detection rate of depression among freshmen is significantly higher than that of the general college student population (6). Therefore, exploring the development and mechanism of depression among freshmen is crucial for the effectiveprevention and treatment of mental health issues among university students.

### 1.1 Negative life events and depression

A large number of studies show that family environment factors, social and cultural factors, individual cognitive factors, biological genetic factors, personality traits, and other factors play different roles in the occurrence and development of depression (7). Among many factors, the predictive effect of negative life events on depression attracts the attention of many scholars (8, 9). Negative life events are defined as “events that can lead to maladjustment and disturbances that most likely result in readjustment-requiring changes in one's daily life” (10), such as poor academic performance, traffic accidents, natural disasters, interpersonal problems, conflicts with parents and so on (8). Studies find that negative life events are the significantly proximal predictors of depression among adolescents (11). Academically, university freshmen may encounter negative events such as the absence of clear learning goals, inadequate academic planning, and difficulties with college courses. Interpersonally, negative events include family conflicts, roommate tensions, and peer pressure. Additionally, Challenges in adjusting to campus life, such as unfamiliarity with campus activities, can also lead to negative effects (12). Previous studies have shown that negative life events are significantly associated with an increased risk of depression (13, 14). However, most of the studies are cross-sectional studies and it is difficult to provide evidence of causal relationships. Therefore, by using longitudinal design, this research explores the relationship between negative life events and depression among university freshmen over time.

It is worth noting that not every individual will experience depression in the face of negative life events. Depression is explained by multiple different risk factors that have probabilistic risk effects; no single risk factor is necessary or sufficient to explain its etiology (7). The diathesis-stress model of depression believes that stress, diathesis and vulnerability jointly stimulate the development of depression (15). Individual vulnerability is activated under stress or negative life events, which results in depression. The vulnerability or protective factors of depression mainly help keep a person from developing mental ill-health or not, such as family or peer support, or other environmental influences (16). However, a reasonable explanation still lacks as to how negative life events, as an external factor, affect freshmen's depression and what kind of individual qualities can buffer against depression.

### 1.2 The mediating role of perceived social support

Perceived social support, defined as an individual expectation and evaluation of social support (17), is a key concept in the social support structure. Essentially, it is an individual satisfaction and emotional experience of being supported, respected, and understood during social interaction (18). Research shows that compared with actual social support, perceived social support acting as a cognitive filter has a stronger effect on individual mental health; perceived social support is more significant in predicting an individual's mental health (9, 19). According to the stress-buffering effect model of social support (20), under a stressed condition, social support can buffer the impact of negative life events on individuals to a certain extent, thus promoting their good social adaptation. Many studies show that perceived social support is significantly negatively related to negative life events and can prevent the negative effects of stresses on mental health and promote better mental health of individuals (19, 21). Moreover, perceived social support has a negative predictive effect on depression. Compared with those with higher perceived social support, college students with lower perceived social support have a higher risk of developing depression symptoms (22). Therefore, negative life events may affect freshmen's depression through perceived social support.

In addition, the mediating process of negative life events affecting college students' depression through perceived social support may vary due to individual differences. Therefore, this study examines the impact of additional factors on this process, aiming to provide a more comprehensive understanding of the underlying mechanisms of depression.

### 1.3 The moderating role of self-esteem

Self-esteem is a subjective measure of the relationship between individuals and society and important others (e.g., parents, spouses, or peers), which reflects whether individuals have good interpersonal relationships and what kind of emotional experience they have (23). As an important individual internal resource, self-esteem plays a critical role in protecting the healthy growth of adolescents (24). The sociometric theory of self-esteem derived from evolutionary psychology and symbolic interaction theory points out that self-esteem is like the gauge of individual interpersonal relationship, which can regulate the process of individual cognition and evaluation of external people and things (25). Studies have shown that individuals with high self-esteem have better social relationships and a better sense of control over their environment, who can filter out negative information, maintain a positive state of mind in the face of various life events (26, 27). However, high self-esteem may be a cause of negative outcomes (28, 29). With the increasing of negative life events, such as punishment and loss, individuals' negative evaluation of themselves and others is rapidly activated (30). By contrast, individuals with low self-esteem are pessimistic and negative in their understanding and explanation of themselves and various daily events in life (23), and they also have poor interpersonal relationships with others (26). They often fail to get respect, understanding, and support for a long time (25), resulting in very limited social support they feel. Due to their negative cognition, even with the increasing of negative life events, their perceived level of social support does not undergo significant changes. In other words, self-esteem moderates the relationship between negative life events and perceived social support.

### 1.4 Present study

**Figure 1:**
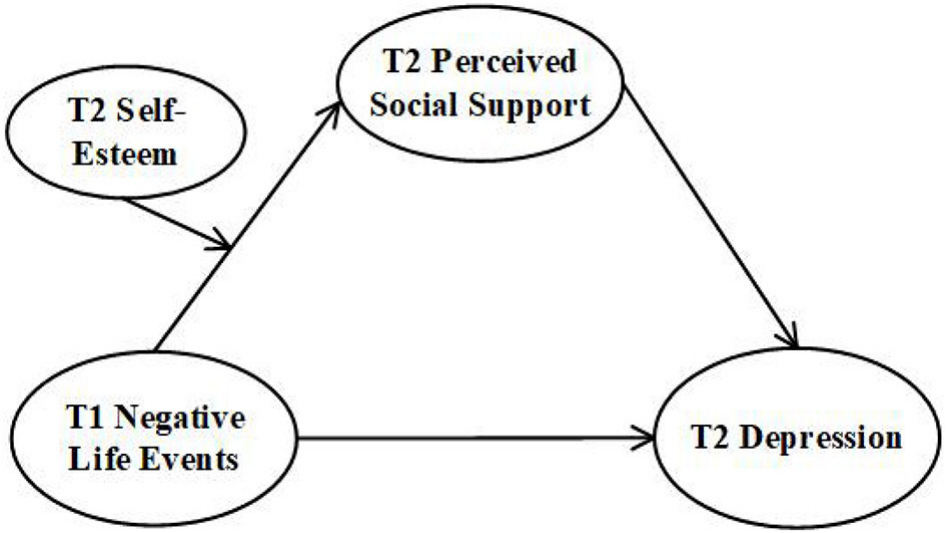
The proposed research model.

Based on the above reasoning, the current study aims to aims to explore the long-term effect of negative life events on depression in university freshmen, and the potential mediating role of perceived social support and the moderating role of self-esteem. The following three hypotheses were proposed, as illustrated in Figure 1.

**Hypothesis 1**. Freshmen's experiences of negative life events positively predict later depression over time.

**Hypothesis 2**. Perceived social support plays a mediating role between negative life events and freshmen's depression.

**Hypothesis 3**. Self-esteem plays a moderating role between negative life events and perceived social support.

## 2 Materials and methods

### 2.1 Participants

The participants were freshmen, aged 18–22, from a university in Hunan Province, China. They were followed up at two-time points and had sufficient reading ability to complete four questionnaires. The time points of the first and the second surveys were recorded as T1 (October 2023) and T2 (May 2024), respectively. A total of 827 questionnaires were distributed for the first time, and 811 questionnaires were obtained for the second time. Suffering from any diagnosed psychopathological disorder, undergoing psychological or psychiatric therapy, or presenting an uncompleted self-report questionnaire were all reasons for exclusion. After matching the data of the two questionnaires, 785 effective questionnaires participated in the two follow-up surveys, with a loss rate of 5.08%. The final sample consisted of 785 participants, with 55.92% female. A total of 53.37% of the participants were from urban areas, and the rest were from rural areas. Moreover, the distribution of freshmen across academic disciplines was relatively balanced, with 34.3% in the humanities, 35.3% in science, and 30.4% in the arts. The mean age of participants in the first survey was 18.85 years (SD = 0.73), and in the second survey was 19.48 years (SD = 0.85).

### 2.2 Procedure

This study was approved by the Human Research Ethics Committee of the corresponding authors' institution. All research procedures follow the Code of Ethics for Clinical and Counseling Psychology of the Chinese Psychological Society. Questionnaires were administered in the middle of each of two consecutive semesters. The researchers obtained prior approval from the department director at the corresponding author's institution to use a classroom and scheduled a convenient time for participants to complete the questionnaires. The participants were informed by well-trained psychology postgraduate students about the purpose of the study and voluntary participation. In addition, the participants were informed that they had the right to withdraw at any time. Furthermore, they were assured that the data would remain strictly confidential, with completed questionnaires were only available to the research team. After signing the written consent, each participant received paper-and-pencil questionnaires and spent ~20 min to complete them.

### 2.3 Measures

#### 2.3.1 Adolescent self-rating life events checklist

Adolescents rated their own negative life events on the Adolescent Self-Rating Life Events Checklist (ASLEC) (31). Liu et al. compiled the scale by combining the physiological and psychological characteristics of adolescents and their social and family roles (32). This scale is used to assess the frequency of life events and the corresponding stress intensity of adolescents. The ASLEC consists of 27 items, which are mainly composed of negative life events and divided into six factors: interpersonal relationship, learning stress, punishment factor, loss factor, health adjustment factor, and other factors. Examples of items include the following: “To be misunderstood or misjudged by others,” “There are contradictions within the family,” and “a heavy load of study.” In this study, adolescents were asked to read each event and rate it on a 5-point scale (1 = no impact, 5= strongly/extremely heavy impact) if the event had happened to them in the last 3 months. Mean scores on the items were calculated, and the higher the score, the higher the level of the negative psychological effect of the life events. In the current study, the Cronbach's alpha coefficients for the ASLEC at T1 and T2 were 0.95 and 0.93, respectively.

#### 2.3.2 Perceived social support scale

Perceived social support was measured by the Chinese version of the Perceived Social Support Scale (PSSS). This scale was compiled by Zimet et al. (17) and revised by Jiang (31). All participants recruited in this study were college students. Referring to previous studies, the “leaders, relatives and colleagues” in the original scale changed to “teachers, classmates, and relatives” (33). This scale includes 12 items. A sample item is: “There are people (teachers, classmates, and relatives) with whom I have been able to share happiness and sorrow.” Adolescents rated each item on a 7-point scale (1 = strongly disagree, 7 = strongly agree). Mean scores on a total of 12 items were calculated, and the higher score corresponds to a higher level of perceived social support. In the current study, the Cronbach's alpha coefficients for the PSSS at T1 and T2 were 0.94 and 0.93, respectively.

#### 2.3.3 Self esteem scale

Adolescents' self-esteem was measured by the Self Esteem Scale (31), which has 10 items. A representative item is: “I feel that I am a valuable person, at least on the same level as others.” This scale scores from 1 = never to 4 = always, with a higher score indicating stronger self-esteem. Mean scores on a total of 10 items were calculated. In the current study, the Cronbach's alpha coefficients for this scale at T1 and T2 were 0.87 and 0.87, respectively.

#### 2.3.4 Self rating depression scale

Depression was measured by the Self Rating Depression Scale (31), which includes 20 items. A sample item is: “I felt weak and tired easily.” Adolescents rated each item on a 4-point scale (1 = no or very little time, 4 = most or all of the time). Mean scores on a total of 20 items were calculated, with higher scores indicating higher levels of depression. In the current study, the Cronbach's alpha coefficients for this scale at T1 and T2 were 0.82 and 0.80, respectively.

### 2.4 Data analysis

Statistical Package for Social Sciences (SPSS-V.25.0) was used to analyze the data. All models were tested in Mplus version 8.0 (34). First, we developed a mediating model to investigate the impact of negative life events on depression and the mediating role of perceived social support in this relationships. Then, a moderation model was established to further examine the moderating effect of self-esteem on negative life events and perceived social support. We applied Bias-Corrected Bootstrap (BC) in the bootstrapping procedure (35) with 5,000 subsamples to estimate the direct and indirect effects in our conceptual model. Bootstrapping is more powerful than the method of Baron and Kenny (36) to check the mediation effect, as it is unnecessary to assume that samples are of normal distribution and it also can obtain a more confident estimation of the indirect effect (37). If the 95% confidence interval (CI) does not contain zero, then the direct and indirect effects are significant. **Table 4** presents the results of the bootstrapping analysis after we controlled for the effects of gender and family socioeconomic status. The main variables in the mediation model and the mediated moderation model were standardized and the analyses were all based on *Z*-scores.

## 3 Results

### 3.1 Common method deviation test

According to the suggestion of Podsakoff and Organ (38), Harman single factor test was used to check whether there was a common method deviation. The results suggested that the explanatory capacity of the first principal component variation extracted by non-rotating factors was 18.81%, which was less than the standard threshold of 40%, and 14 factors with characteristic values >1 were separated. Therefore, common methodological biases were excluded in this study.

### 3.2 Descriptive statistics

Table 1 revealed that T2 depression was significantly negatively correlated with T2 perceived social support (*r* = −0.38, *p* < 0.01) and T2 self-esteem (*r* = −0.65, *p* < 0.01). In addition, T2 depression was significantly positively correlated with T1 negative life events (*r* = 0.17, *p* < 0.01). Furthermore, T2 perceived social support was positively correlated with T2 self-esteem (*r* = 0.39, *p* < 0.01) and negatively correlated with T1 negative life events (*r* = −0.06, *p* < 0.01). Lastly, T2 self-esteem was negatively correlated with T1 negative life events (*r* = −0.12, *p* < 0.01).

**Table 1:** Descriptive statistics and correlations for the main variables.

**Variables**	**1**	**2**	**3**	**4**	**5**	**6**	**7**	**8**	**9**	**10**
1. Gender	1.00									
2. SES	—	1.00								
3. T1 depression	0.002	−0.02	1.00							
4. T2 depression	0.06	−0.07	0.38^**^	1.00						
5. T1 perceived social support	0.02	0.02	−0.39^**^	−0.19^**^	1.00					
6. T2 perceived social support	−0.01	0.01	−0.26^**^	−0.38^**^	0.43^**^	1.00				
7. T1 self-esteem	−0.01	−0.04	−0.62^**^	−0.35^**^	0.42^**^	0.26^**^	1.00			
8. T2 self-esteem	−0.02	0.004	−0.34^**^	−0.65^**^	0.22^**^	0.39^**^	0.50^**^	1.00		
9. T1 negative life events	0.06	0.01	0.33^**^	0.17^**^	−0.14^**^	−0.06^**^	−0.19^**^	−0.12^**^	1.00	
10. T2 negative life events	0.10^**^	0.06	0.19^**^	0.29^**^	−0.09^**^	−0.19^**^	−0.16^**^	−0.19^**^	0.28^**^	1.00
*M*	—	—	1.80	1.89	5.68	5.40	3.07	3.04	1.63	1.72
SD	—	—	0.30	0.34	0.74	1.01	0.37	0.47	0.35	0.58

SES, socioeconomic status.

^**^p < 0.01.

### 3.3 The mediating effect of perceived social support

Referring to the test method proposed by Wen and Ye (39) and Mackinnon (40), the present study investigated the impact of negative life events on depression, the mediating role of perceived social support in the above relationship, and the moderating role of self-esteem (Figure 1). All variables were standardized.

Model 1 was constructed to test the direct effects of negative life events and perceived social support on depression, and gender and family socioeconomic status were taken as control variables. As shown in Table 2, the fit indicators according to the model were: AIC = 6335.52, χ^2^ = 137.90, df = 26, χ^2^/df = 5.30, CFI = 0.96, TLI = 0.95, RMSEA = 0.07, SRMR = 0.04, and the fitting of the model was acceptable. The results indicated that negative life events significantly positively predicted the depression level of freshmen, and the direct effects were: β = 0.12, *p* < 0.01. H1 was verified.

**Table 2:** Model fit index for all structural equations.

**Model**	**χ^2^**	**df**	**χ^2^/df**	**AIC**	**CFI**	**TLI**	**RMSEA**
Model 1	137.90	26	5.30	6,335.52	0.96	0.95	0.07
Model 2	178.23	48	3.71	12,223.12	0.97	0.95	0.06
Model 3	487.74	60	8.13	18,169.59	0.90	0.90	0.08

Model 1, direct effect (negative life events → depression); Model 2, The mediating model; Model 3, The moderated mediation model.

**Figure 2:**
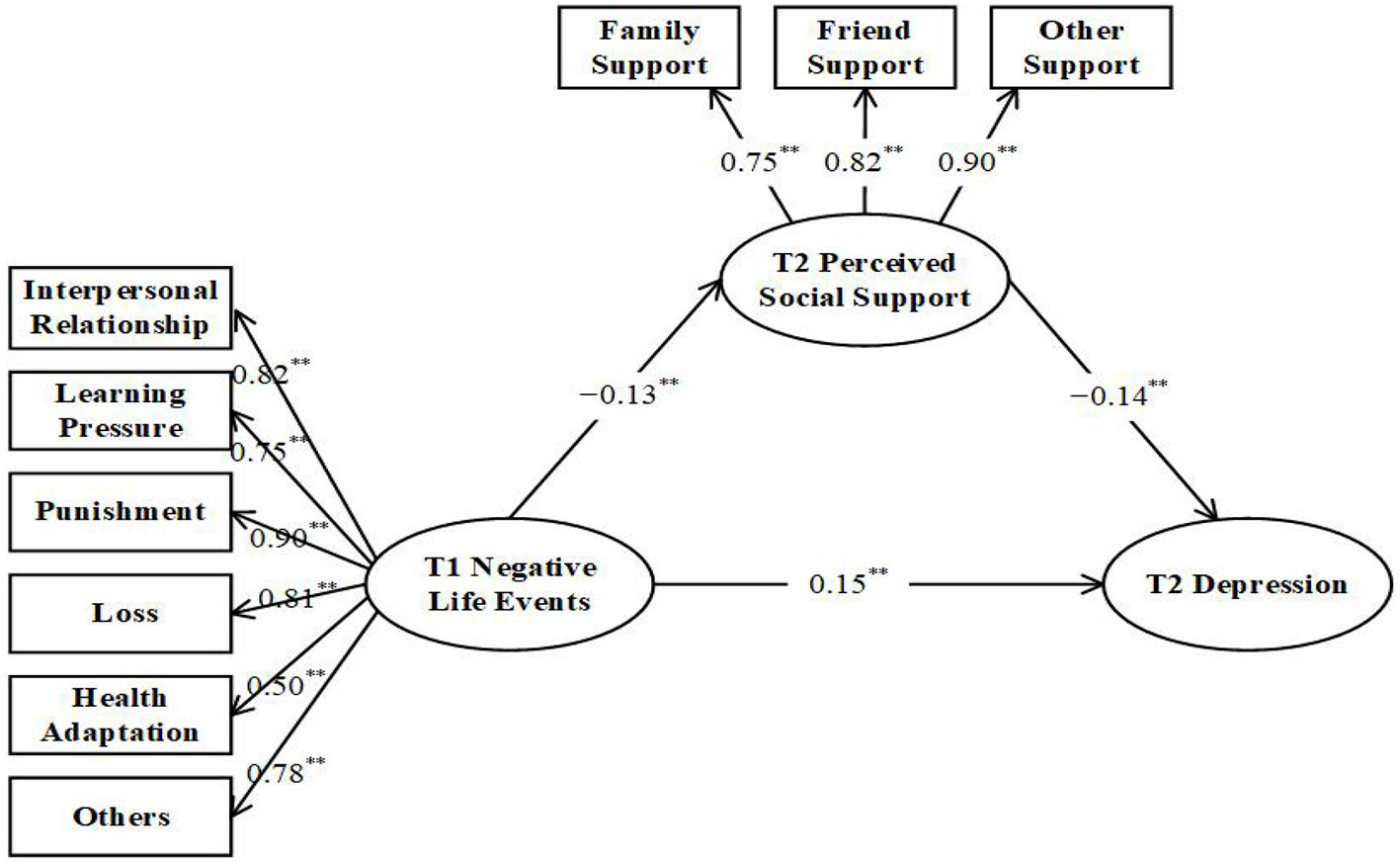
Mediating model. The values in the figure are standardized path coefficients, the same below, **p < 0.01.

To test whether or not perceived social support mediates the relationship between negative life events and depression, a structural equation model (Model 2) was constructed with negative life events as the predictive variable, perceived social support as the intermediary variable, and depression as the outcome variable (Figure 2). After testing, all fitting indicators of the model were good after controlling gender and family socio-economic status, AIC = 12223.12, χ^2^ = 178.23, df = 48, χ^2^/df = 3.71, CFI = 0.97, TLI = 0.95, RMSEA = 0.06, SRMR = 0.04 (see Table 2). The specific path coefficient was shown in Table 3. According to the suggestion of Preacher and Hayes (35), 5,000 samples were taken to test by using the Bias-Corrected Bootstrap method. Perceived social support played a partial mediating role between negative life events and depression, β_ab_ = 0.02, SE = 0.01, 95% CI = (0.01, 0.03). The ratio of intermediary effect to total effect was 0.02/0.17 = 0.12, so the proportion of intermediary effect was 12%. H2 was validated.

**Table 3:** Summary of path coefficients and mediating effects tests.

**Dependent variable**	**Model paths**	**Coefficient (β)**	**SE**	**Bootstrap95%**	
				**LLCI**	**ULCI**
Depression	**Direct effect**	0.15	0.02	0.12	0.15
	Negative life events → depression				
	**Indirect effect:**	0.02	0.01	0.01	0.03
	Negative life events → self-esteem → depression				
	**Total effect:**	0.17	0.03	0.11	0.16
	Negative life events → depression				
	*R* ^2^	0.01			

### 3.4 The moderated mediation model

**Figure 3:**
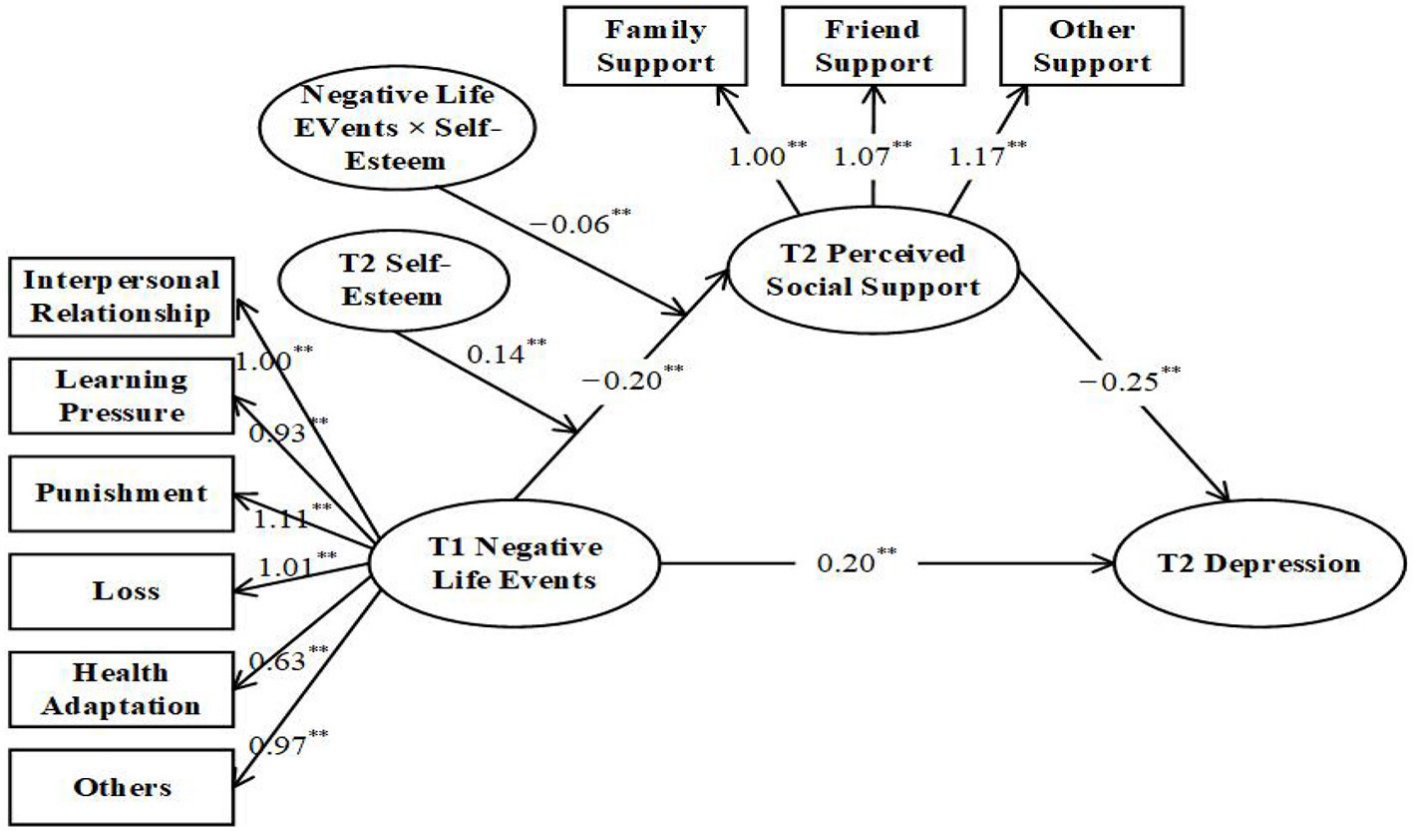
Full moderated mediation model. **p < 0.01.

Then, a moderated mediated model (Model 3) was established, which included self-esteem, the interaction items of self-esteem, and the negative life events. At first, the paths of regulatory effect of self-esteem were tested. The regulatory effect of self-esteem on the “a” path (the relationship between negative life events and perceived life support) was tested, followed by the “b” path (the relationship between perceived social support and depressive symptoms) and the “c” path (the direct relationship between negative life events and depressive symptoms). The test results revealed that self-esteem had no significant regulatory effect on the “b” path and “c” path (the interaction items: β = 0.005, SE = 0.01, *p* = 0.60; β = −0.006, SE = 0.01, *p* = 0.51). The model fitting results after including self-esteem in the “a” path were as follows: AIC = 18169.59, χ^2^ = 487.74, df = 60, χ^2^/df = 8.13, CFI = 0.90, TLI = 0.90, RMSEA = 0.08 (see Table 2). The specific path was shown in Figure 3 and Table 3. The bootstrap test results demonstrated that the interaction between negative life events and self-esteem had a significant predictive effect on perceived social support (β = −0.06, *p* < 0.01) (see Table 4). The mediating effect of perceived social support was significant, β_ab_ = 0.05, SE = 0.02, 95% CI = (0.02, 0.10), and the amount of mediating effect was 20%. There was a negative correlation between negative life events and perceived social support, so the effect of interaction items on perceived social support was also negative. This outcome indicated that a higher negative correlation between negative life events and perceived social support would become as the self-esteem increased. The results of the moderate mediated model analysis found that self-esteem enhanced the negative effect of negative life events on perceived social support. H3 was verified, and self-esteem moderated the “a” path.

**Table 4:** The results for the mediated moderation models.

**Variable**	**Coefficient (β)**	**SE**	***t***
Gender	0.12	0.06	1.83
Family socioeconomic status	−0.06	0.03	−1.80
T1 negative life events	0.20	0.03	4.13^**^
T2 self-esteem	0.14	0.04	−3.02^**^
T2 perceived social support × T2 self-esteem	−0.06	0.04	−1.66^**^
T2 perceived social support	−0.25	0.03	2.79^**^
*R* ^2^	0.03		

^**^p < 0.01.

**Figure 4:**
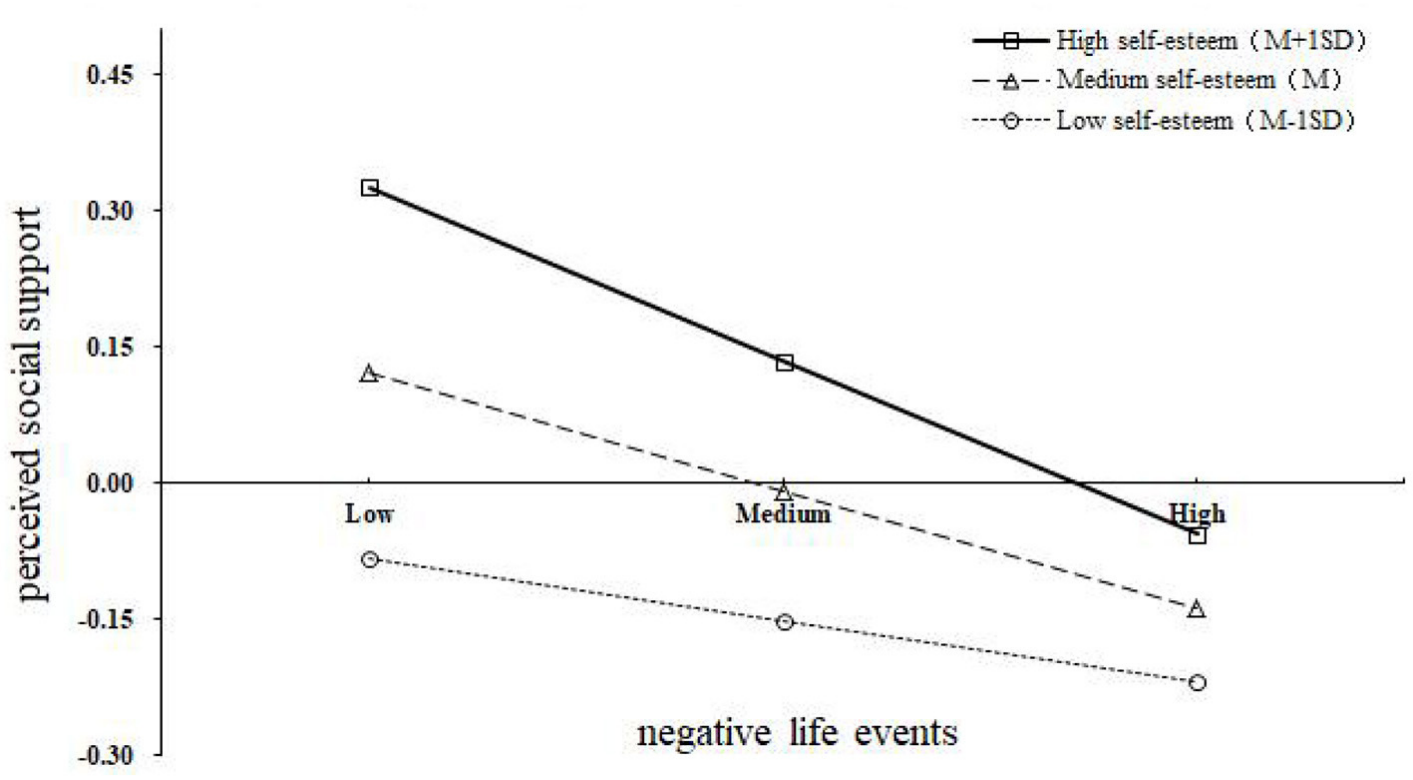
Interactive effect of negative life events at T1 and self-esteem at T2 on perceived social support at T2.

To analyze how self-esteem moderates the mediating effect of perceived social support on the negative life events and depression of university freshmen in detail, this study took two levels of self-esteem: one standard deviation higher than the average and one standard deviation lower than the average (41). A simple effect test was performed, and an interaction effect diagram was drawn (Figure 4). Under the condition of low self-esteem, the correlational coefficiency between negative life events and perceived social support was β = −0.06, SE = 0.04, *p* < 0.01, 95% CI (−0.14, 0.03), and the mediating effect of perceived social support was β_ab_ = 0.02, SE = 0.01, 95% CI (0.03, 0.20). Under the condition of high self-esteem, the correlational coefficiency between negative life events and perceived social support was β = −0.21, SE = 0.01, *p* < 0.01, 95% CI (−0.32, −0.10), and the mediating effect of perceived social support was β_ab_ = 0.03, SE = 0.01, 95% CI (0.05, 0.24) (see Table 4). With the increase in self-esteem, the correlation coefficiency between negative life events and perceived social support became higher, and perceived social support played a stronger intermediary role between negative life support and depression.

## 4 Discussion

Theories and empirical studies alike indicated that negative life events can serve as an external stress factor that significantly predicts the development of college students' depression (6, 42). However, the protective factors and mechanisms through which negative life events longitudinally predicted depression are not fully understood. This study addressed these research gaps by using a longitudinal design among a sample of Chinese college students. This study examined the mediating effect of perceived social support in the link between negative life events and depression and investigated the moderating roles of self-esteem in the links between negative life events and depression via perceived social support. Supporting the hypotheses, the results highlighted that university freshmen's negative life events could predict an increase in their depression over time. Additionally, perceived social support partially mediated the longitudinal relationship between negative life events and depression. The results also revealed that self-esteem enhanced the negative effect of negative life events on perceived social support. Moreover, with a high level self-esteem, the mediating effect of perceived social support on the longitudinal relation between negative life events and depression was enhanced.

The current study found that negative life events significantly predict the depression of freshmen 6 months later, even after controlling the baseline depression. These findings align with previous cross-sectional research, which has consistently shown a significant association between university students' negative life events experiences and concurrent depression (43). Notably, the current study suggests that negative life events may not only lead to immediate depressive symptoms but are also prospectively associated with depression 6 months later. Therefore, it would be beneficial to implement early interventions aimed at mitigating the long-term impact of these negative events on mental health.

The mediation effect analysis suggested that negative life events not only predict university students' depression but also affect depression through perceived social support. This finding is consistent with previous research results (11, 21, 44). Negative life events positively predict the depression of university freshmen, indicating that the occurrence of negative life events has a negative impact on the mentality of university freshmen to a certain extent. The occurrence of negative life events reduces the subjective wellbeing of individuals and puts them in a sub-health state (45, 46). The occurrence of negative life events can also induce negative emotions, such as anxiety and loneliness (47, 48). Another research study indicated that negative life events lead to depression and even suicide (49). Perceived social support, as a protective factor against depression, is an important intermediary factor in the process of individual depression caused by negative life events (44). On the one hand, the more negative life events an individual experiences, the lower the level of perceived social support (21). On the other hand, individuals with low levels of perceived social support have a higher risk of inducing depression (5). Perceived social support is an important predictor of depression (19). Individuals with high levels of perceived social support are more likely to feel respected, supported, and understood by others. When faced with negative life events, perceived social support can effectively reduce the risk of developing depression among individuals (9, 18). Hence, enhancing perceived social support among university freshmen may help mitigate the risk of depression.

In addition, self-esteem significantly moderated the first half of the mediating effect of perceived social support, but not the direct path. This finding indicated that the mediating effect of perceived social support was affected by freshmen's level of self-esteem, whereas the direct predictive effect of negative life events on freshmen's depression was not affected by individuals' level of self-esteem. This result partially supports relevant previous research results (27). Specifically, comparing to university freshmen with high self-esteem, with the increase of negative life events, the perceived social support level of individuals with low self-esteem declines more slowly. Brown et al. found that self-esteem has two important psychological functions, namely, self-enhancement (expansion) and self-protection (buffering) (30). Initially, individuals with high self-esteem tend to have positive social experiences and strong social relationships, while those with low self-esteem are full of negative self-perception and less social engaged (23, 26). However, as negative life events accumulate, such as punishment, loss of important others, interpersonal conflicts, or academic pressure, the reactions of these two groups diverge. Individuals with low self-esteem are less susceptible to the impact of various negative life events due to their low self-concept and self-efficacy, leading to a slower decrease in perceived social support. In contrast, individuals with high self-esteem, in order to maintain their good self-image and social relationships, may hesitate or feel ashamed to engage in social activities after experiencing negative life events, resulting in a sharp decline in perceived social support. However, due to the fact that individuals with low self-esteem initially experience lower levels of external support, although individuals with high self-esteem experience a significantly faster decline in their perceived social support than individuals with low self-esteem, the average perceived social support of individuals with high self-esteem is still higher than that of individuals with low self-esteem. In sum, our findings contribute to the nuanced understanding of self-esteem's moderating role and offer insights for developing targeted interventions: for low self-esteem individuals, interventions should focus on enhancing social support networks; for high self-esteem individuals, strategies could involve teaching coping and emotional regulation skills to reduce sensitivity to social changes.

## 5 Implication and limitations

The current study make significant theoretical and practical implications. On the one hand, this study contributes to the literature by employing a longitudinal design to examine the relationship between negative life events and depression, along with its mechanisms. Using perceived social support as a mediator and self-esteem as a moderator, it clarifies how negative life events influence depression over time and the conditions under which they have a stronger impact. Second, focusing on freshmen, a high-risk group for psychological issues like depression, provide valuable insights into this vulnerable population within the university context. On the other hand, the findings provide evidence for developing mental health interventions targeting university freshmen. For example, it is essential to establish prevention programs for negative life events. Moreover, this study offers valuable reference for depression interventions among freshmen in regions with similar cultural contexts to China.

Like any research, this study also has some limitations. First, the study utilized self-report questionnaires, which may decrease the internal validity of the results. Although the bias effect caused by the common method was minimal, we recommend that future research incorporate observational data as supplementary. Second, the study collected data within 6 months intervals, which limits understanding into the effects of negative life events on depression over a longer period. Future research could benefit from multiple measurements across extended time period to develop a more comprehensive understanding.

## 6 Conclusion

Using longitudinal research method recruiting 785 Chinese university freshmen, this study established a moderate mediating model between negative life events and depression. The results revealed that: (1) negative life events had a negative impact on depression; (2) perceived social support partially mediated the relationship between negative life events and depression; (3) self-esteem moderated the relationship between negative life events and perceived social support. These findings have significant implications on reducing the risk of depression and promoting mental health among university freshmen.

## Data Availability

The raw data supporting the conclusions of this article will be made available by the authors, without undue reservation.
